# Supplementary material for: Ocean colour signature of climate change
Source: Nat Commun. 2019 Feb 4;10:578. doi: 10.1038/s41467-019-08457-x (PMC6362115; doi:10.1038/s41467-019-08457-x)
Supplement: Supplementary file 1 — Supplementary Information [file 41467_2019_8457_MOESM1_ESM.pdf]

SUPPLEMENTARY MATERIAL

for

Ocean Colour Signature of Climate Change

Dutkiewicz et al

SUPPLEMENTARY FIGURES:

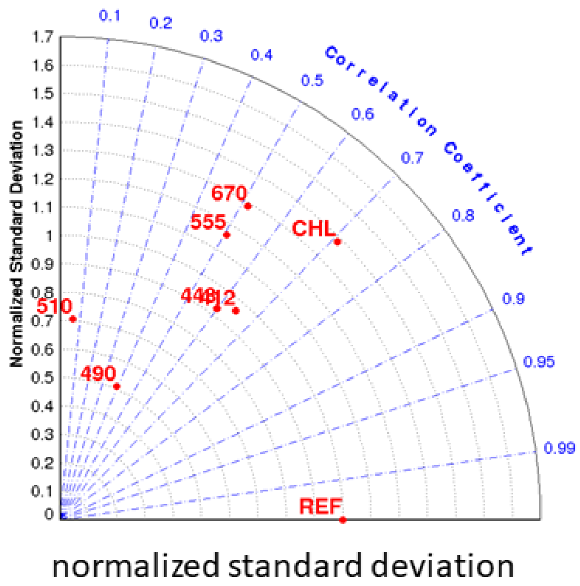

<u>Supplementary Figure 1</u>: Taylor diagrams of model global composite relative to OC-CCI products (1998-2015). *This polar coordinate plot shows correlation (angular position) and the normalized (by observed spatial STD) spatial standard deviation (radial position). Statistics are performed on log-normalized fields. REF indicates a perfect match between model and observations. CHL refers to the model derived Chl-a, and the numbers refer to the model R_{RS} interpolated to the same wavebands as the OC-CCI products (412, 443, 490, 510, 555, 670nm). This interpolation is problematic in the 490 and 510nm bands where the patterns of high/lows shift between those seen in the blue and the green (see Supplementary Figure 2), and likely skew the results here.*

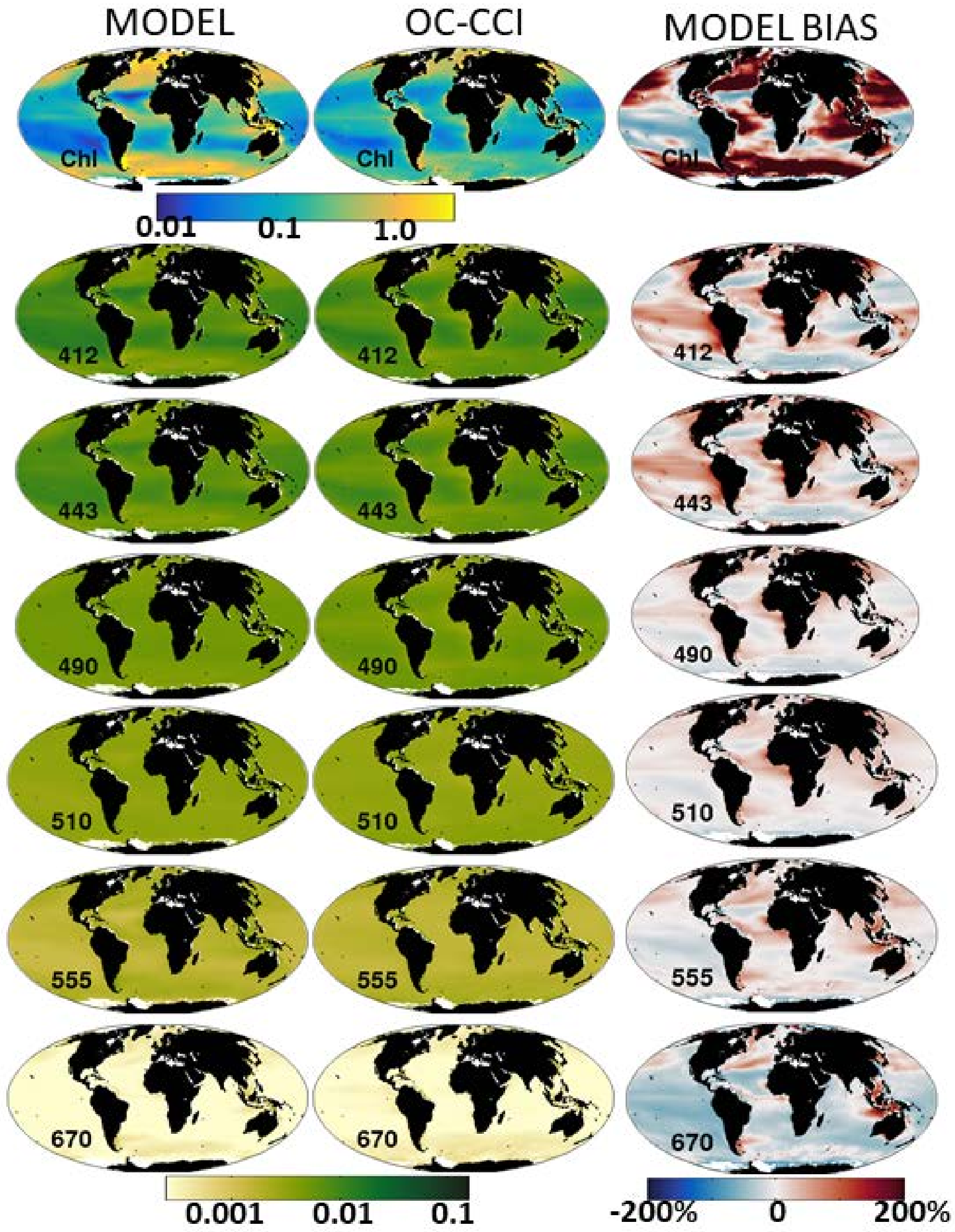

<u>Supplementary Figure 2.</u> Comparison of 18 year mean composites (1998-2015). *Left column is model, middle column is OC-CCI products and right column is the % bias. "Chl" refers to the model "derived" Chl-a, and the numbers refer to the model R_{RS} interpolated to the same wavebands as the OC-CCI products (412, 443, 490, 510, 555, 670nm). Chl-a in left and middle columns has units of mg Chl m^{-3}, R_{RS} has units of sr^{-1}.*

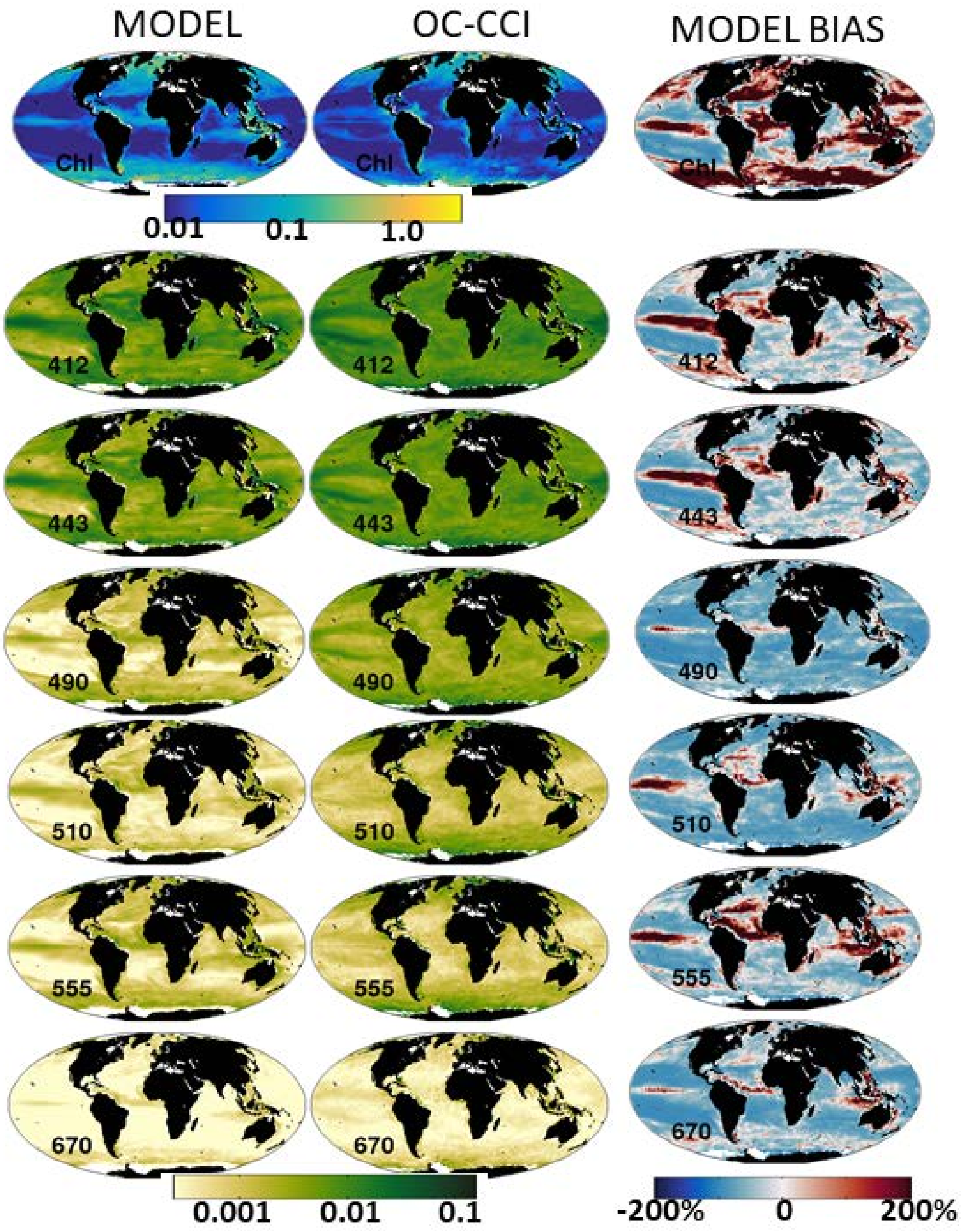

***Supplementary Figure 3.* Comparison of 18 year annual variability (1998-2015).** *Shown are the temporal standard deviation of the de-trended, log-normalized annual composites over the 18 year study period. Left column is model, middle column is OC-CCI products and right column is the % bias. "Chl" refers to the model "derived" Chl-a, and the numbers refer to the model R_{RS} interpolated to the same wavebands as the OC-CCI products (412, 443, 490, 510, 555, 670nm). Chl-a in left and middle columns has units of mg Chl m^{-3}, R_{RS} has units of sr^{-1}.*

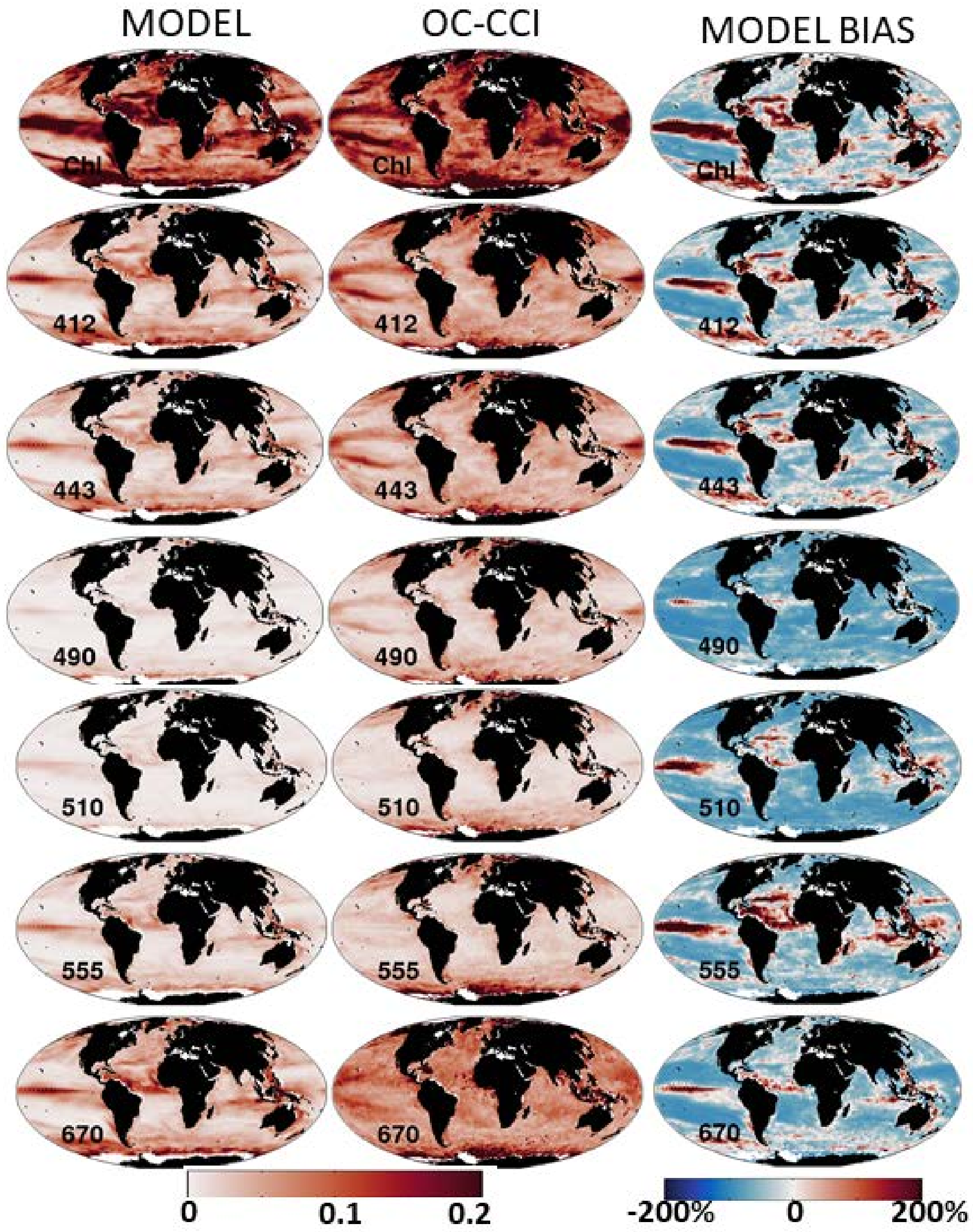

***Supplementary Figure 4.* Ratio of interannual variability to the climatological composite mean (1998-2015).** *Shown are the ratio of the temporal standard deviation of the de-trended, log-normalized annual composites over the 18 year study period to the 18 year mean composite. Left column is model, middle column is OC-CCI products and right column is the % bias. "Chl" refers to the model "derived" Chl-a, and the numbers refer to the model R_{RS} interpolated to the same wavebands as the OC-CCI products (412, 443, 490, 510, 555, 670nm). Left and middle columns are unitless ratios. Global median values are shown in Fig 3.*

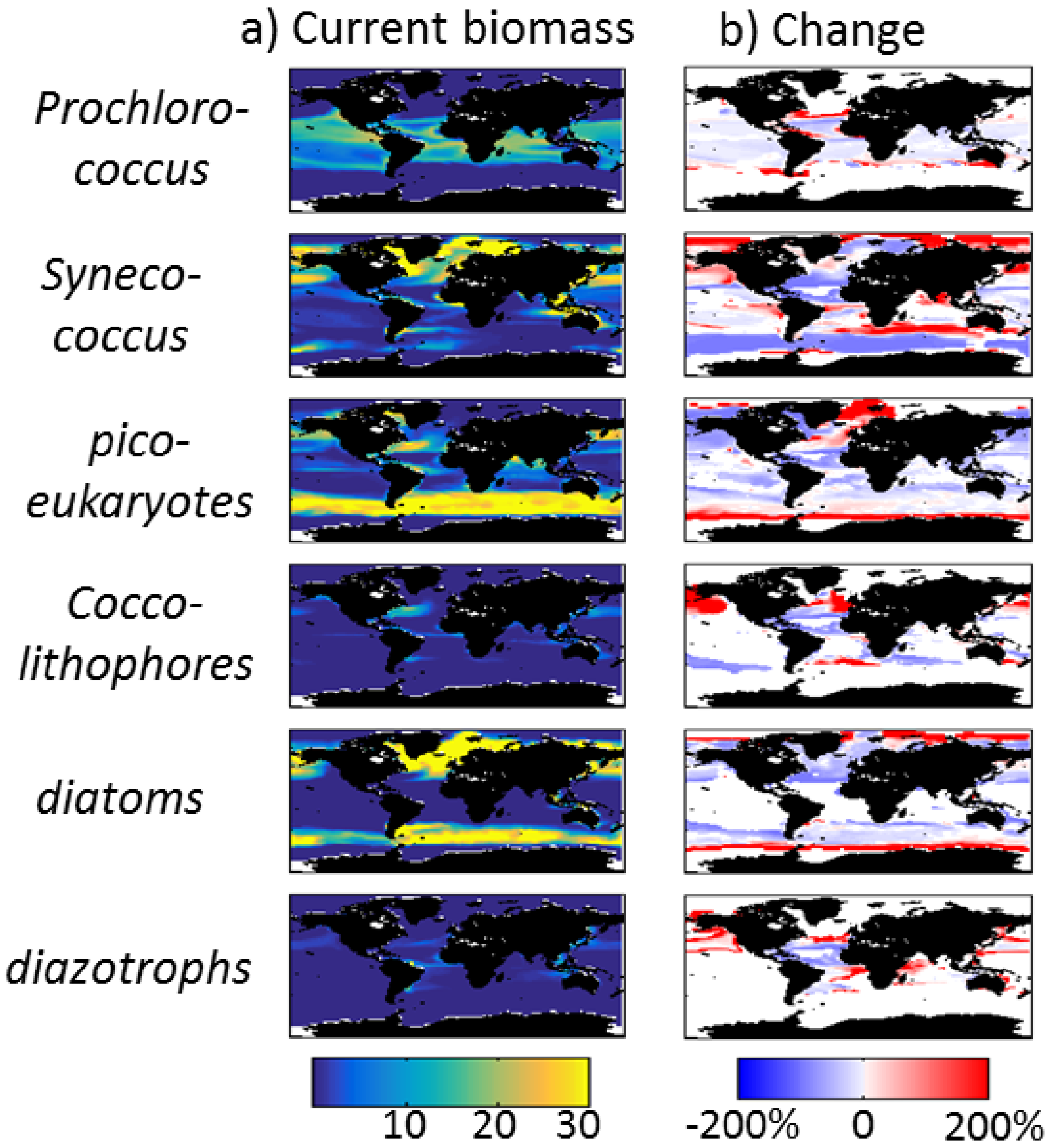

***Supplementary Figure 5.* Model phytoplankton types.** *(a) Left panels show the 1998-2015 mean of the phytoplankton types biomass (mgC/m^3). (b) Right panels show the biomass difference (mgC m^{-3}) between the 2085-2100 mean and the current day mean (i.e. the left panel). Areas in (b) where the differences between the two periods are not significant (p>0.05) or where the particular phytoplankton group biomass is below a critical threshold are left blank.*
